# Eptacog Alfa (Activated) Is Physically and Chemically Stable over 24 Hours when Administered as Bolus Injections in an Automated Infusion Pump

**DOI:** 10.1055/s-0039-1678684

**Published:** 2019-02-06

**Authors:** Per Rexen, Jane Taaftegaard Jensen, Nina Bjorn Schwerin, Elena Kozina

**Affiliations:** 1 Novo Nordisk A/S, Novo Allé, Bagsvaerd, Denmark

**Keywords:** rFVIIa, eptacog alfa (activated), congenital hemophilia with inhibitors, in-use stability, automated infusion, surgery

## Abstract

**Introduction** Eptacog alfa (activated) is a recombinant activated factor VII (rFVIIa) used for the treatment and prevention of bleeding episodes in patients with congenital hemophilia with inhibitors. Frequent dosing requirements make the use of an automated bolus infusion pump a promising alternative to manual administration.

**Aims** The objective of this in vitro study was to evaluate the physical and chemical stability of room temperature–stable rFVIIa at 25°C over 24 hours in an automated bolus infusion pump.

**Methods** An automated bolus infusion pump with preset bolus injection intervals of 2 to 6 hours was used. Samples of rFVIIa were analyzed for critical quality parameters, presence of leachables, and microbiological growth. The infusion system was evaluated visually.

**Results** rFVIIa is physically and chemically stable when used in an automated bolus infusion pump system for up to 24 hours at 25°C. All critical quality parameter results were within the shelf-life limits and complied with the acceptance criteria. Leachables were observed at concentrations within their respective acceptance criteria. No visual changes in the syringe or infusion tube were observed; inherent particles in the reconstituted rFVIIa similar in size and description to those previously found in rFVIIa were seen. No microbiological growth was detected.

**Conclusions** rFVIIa is stable in a bolus infusion pump system for up to 24 hours at 25°C. Bolus injection intervals of 2 to 6 hours can be used without physical or chemical changes to rFVIIa. This study supports the use of an automated bolus infusion pump in the hospital setting, across all indications for rFVIIa.

## Introduction

Eptacog alfa (activated) (NovoSeven, Novo Nordisk A/S) is a recombinant activated factor VII (rFVIIa) used for the treatment and prevention of bleeding episodes in patients with congenital hemophilia with inhibitors. 1 2

Owing to the short in vivo half-life of rFVIIa, frequent and regular administration is required to maintain hemostasis. The dosing schedule of rFVIIa for congenital hemophilia with inhibitors as per the product label is intravenous bolus injection at 90 µg/kg body weight every 2 to 3 hours. 1 2 The dosing frequency of rFVIIa can make it difficult to maintain the required schedule of reconstitution and administration, due to the time and resources required. Delayed or missed doses, which can inadvertently occur on a busy hospital ward, can lead to impaired hemostasis. 3 For surgical patients in the perioperative period, failure to administer rFVIIa at the designated time intervals may increase the risk of breakthrough bleeding and result in poor patient outcomes. 4

Alternative delivery methods for rFVIIa in the hospital setting for the treatment of serious bleeds and in the perioperative period may be able to improve the convenience associated with dosing of rFVIIa and adherence to a treatment regimen, as well as help circumvent possible treatment errors. Use of an infusion pump to deliver bolus doses of rFVIIa could potentially satisfy these requirements for all rFVIIa indications. For delivery of rFVIIa by bolus infusion to be considered an appropriate option, it must be confirmed that rFVIIa is physically and chemically stable at room temperature (RT) over the time period it is expected to remain in the syringe and administered through an automated pump.

The objective of this study is to evaluate the physical and chemical stability of RT-stable rFVIIa at 25°C over 24 hours in an automated bolus infusion pump.

## Materials and Methods

### Study Design

In this in vitro study, the physical and chemical stability of RT-stable eptacog alfa (activated) was investigated at 25°C over 24 hours when using an automated bolus infusion pump, polypropylene syringe, and infusion tube. Bolus injections with preset intervals from 2 to 6 hours were tested.

### Test Product and Experimental Configuration

**Fig. 1:**
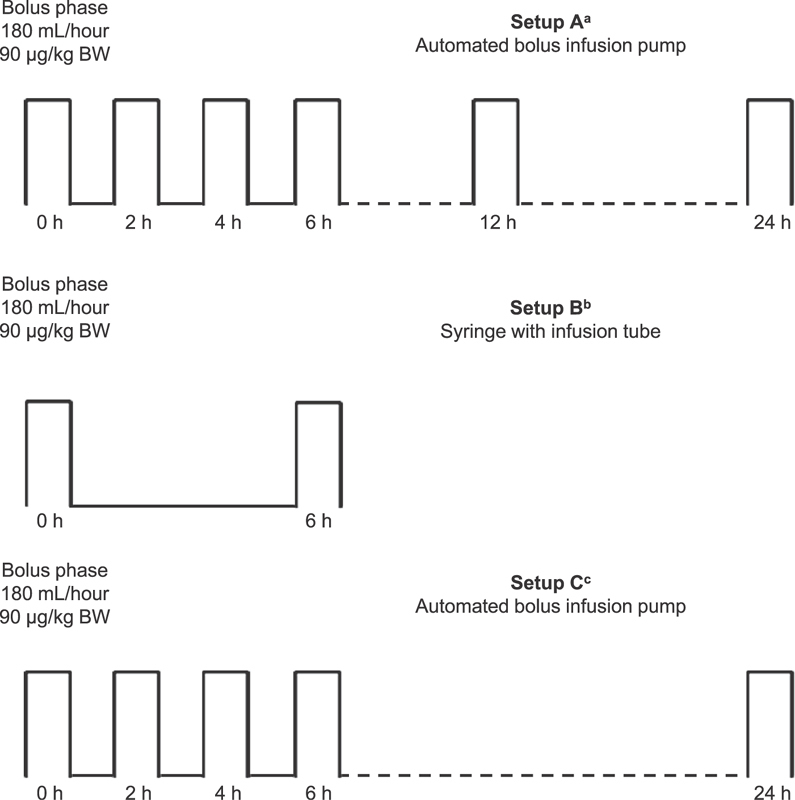
Sampling plan for test systems. All test systems started with initial bolus sample of 2 mL at 180 mL/hour. ^a^ Programmed to deliver 0.01 mL/hour rFVIIa until next bolus sample, to simulate no dosing between bolus injections; ^b^ Tested separately without automated pump, with manual handling of syringe and infusion tube at same rate as automated bolus infusion pump (180 mL/hour); ^c^ Additional batches of rFVIIa tested. As only limited variations were observed between time points in the first part of the study, fewer time points were used; sampling at 12 hours was omitted. BW, body weight; h, hour; rFVIIa, recombinant activated factor VII.

Eptacog alfa (activated) was reconstituted as recommended in the prescribing information 2 with histidine solvent to an rFVIIa concentration of 1.0 mg/mL 1 and transferred to a syringe (BD Plastipak, Becton, Dickinson and Co. Ltd., Ireland). Three batches of rFVIIa were reconstituted to a concentration of 1.0 mg/mL of rFVIIa. The syringe with infusion tube (Original Perfusor Line, Type IV, B. BRAUN Melsungen AG, Germany) and reconstituted rFVIIa was attached to an automated bolus infusion pump (Space Perfusor, Model 8713030, B. BRAUN, Germany). The infusion program was initiated and samples were taken at regular intervals ( Fig. 1 , setups A, B, C). Three different study setups were used; each study setup was run multiple times. Samples were taken from the end of the infusion tube at different time points and were compared with reference samples of rFVIIa. Reference samples of rFVIIa were reconstituted on the day of analysis and stored in the reconstitution vessel. Investigation of product adsorption into the test system was undertaken by comparing degradation of rFVIIa with a reference sample ( Fig. 1 , setup B).

### Assessments of Critical Quality Parameters

Samples of rFVIIa were analyzed for the following critical quality parameters: specific activity, rFVIIa content, aggregates, and degradation products ( Table 1 ). The specific activity of reconstituted rFVIIa was determined by the method described in the European Pharmacopeia. 5 Briefly, samples of reconstituted rFVIIa and reference samples of rFVIIa were incubated at 37°C with factor VII–deficient plasma before adding 40 µL of human tissue factor solution (Dade Innovin, DADE BEHRING). The coagulation time (interval between addition of the human tissue factor solution and the first indication of the formation of fibrin) for the test samples was compared with that of the reference samples and activity in international units (IU) per microgram of rFVIIa calculated. 5 A visual evaluation of the syringe, infusion tube, and reconstituted rFVIIa was performed after storage at 0, 6, 12, and 24 hours at 25°C to observe whether any changes in transparency, color, or sedimentation occurred during 24 hours of use as compared with visual descriptions at the start of the study. The presence of inherent particles, defined in U.S. Pharmacopeia guidance chapter 787 as particles of the protein or formulation components, 6 in reconstituted rFVIIa was also assessed using stereomicroscopy and Fourier transform infrared spectroscopy.

**Table 1:** Analytical parameter results

Analytical parameters		Acceptance limits for difference between analytical results for test and reference a	Batch b	Time points (h) c	Mean difference between test and reference samples	90% confidence limits		Within acceptance limits d
						Lower CI	Upper CI	
Specific activity (IU/μg)		≤8.07	1	6	1.00	–0.16	2.16	Yes
				12	1.17	–0.15	2.48	
				24	–0.83	–1.45	–0.21	
			2	6	2.00	–6.76	10.76	6 h: no
				24	–0.33	–2.91	2.24	24 h: yes
			3	6	0.67	–2.84	4.18	Yes
				24	2.67	–2.20	7.53	
Aggregates	rFVIIa dimers/oligomers (%)	≤3.986	1	6	0.145	–0.059	0.349	Yes
				12	0.082	–0.112	0.276	
				24	0.181	–0.114	0.477	
			2	6	–0.189	–0.443	0.065	Yes
				24	–0.096	–0.283	0.090	
			3	6	0.201	–0.093	0.495	Yes
				24	0.262	–0.013	0.537	
Content of rFVIIa (mg/vial)		≤0.380	1	6	0.064	0.029	0.099	Yes
				12	0.066	0.029	0.103	
				24	0.054	0.032	0.076	
			2	6	–0.055	–0.066	–0.045	Yes
				24	–0.027	–0.081	0.026	
		≤0.747	3	6	–0.078	–0.109	–0.047	Yes
				24	–0.095	–0.161	–0.029	
Degradation products	rFVIIa heavy chain (%)	≤2.736	1	6	0.133	0.112	0.154	Yes
				12	0.170	0.129	0.211	
				24	0.177	0.120	0.233	
			2	6	0.080	–0.058	0.219	Yes
				24	0.152	0.065	0.240	
			3	6	0.068	–0.072	0.209	Yes
				24	0.129	–0.029	0.287	
	rFVIIa oxidized forms (%)	≤1.953	1	6	0.052	0.032	0.072	Yes
				12	0.044	0.027	0.062	
				24	0.074	0.035	0.114	
			2	6	0.043	0.029	0.056	Yes
				24	0.045	–0.037	0.127	
			3	6	0.003	–0.078	0.084	Yes
				24	0.046	–0.027	0.119	

Abbreviations: CI, confidence interval; IU, international unit; rFVIIa, recombinant activated factor VII.

a The value of |DPbolus – FDref| for each analytical setup. 90% confidence limits for the difference must be within the acceptance limits.

b Batch 1 tested in setup A and setup B. Batches 2 and 3 tested in setup C.

c Six replicates per time point.

d All results complied with the shelf-life specification limits.

Samples were analyzed for the presence of leachables migrating from the test system to the drug product using gas chromatography with mass spectrometric detection, high-pressure liquid chromatography with ultraviolet light detection, liquid chromatography with mass spectrometric detection, ion chromatography, and inductively coupled plasma with mass spectrometric detection. The presence of leachables was determined by comparison of chromatograms for reference and test samples. Any potential leachables were quantified and compared with the analytical evaluation threshold limit calculated based on the threshold of toxicological concern and maximum dose volume according to the International Council for Harmonisation of Technical Requirements for Pharmaceuticals for Human Use (ICH) M7 guidelines. 7 For metals and other trace element parameters, the permitted daily exposure was used as the acceptance criteria. 8 9 10

The microbiological holding time of reconstituted rFVIIa was assessed for at least 24 hours. After the 24-hour test period, the samples were inoculated into testing canisters with a suitable culture medium and incubated for 14 days. At intervals during the incubation period and at its conclusion, the canisters were visually examined for macroscopic evidence of microbial growth.

### Statistical Methods

The acceptance criterion was defined to demonstrate compliance with the shelf-life specification at any product age within the shelf life followed by 24 hours in the bolus infusion pump. Therefore, the acceptance interval was calculated as the tolerated change when using a bolus infusion pump for 24 hours that still ensured compliance of the product with the shelf-life specification. The study-specific acceptance criterion for critical quality parameters was defined as a two-sided 90% confidence interval (CI) for the mean difference between results from pump samples taken at different time points and reference samples that must be within the acceptance interval ( Table 1 ).

## Results

All results for the critical quality parameters were within the shelf-life limits and complied with the acceptance criteria ( Table 1 ). The 90% CIs were also within the acceptance intervals for all analyzed batches and time points.

For the parameter specific activity, reference values (taken at 0 hours) were from 50 to 56 IU/µg. Specific activity of rFVIIa samples taken at time points 6, 12, and 24 hours were between 49 and 59 IU/µg. Mean differences between test and reference samples were small and 90% CIs were within the acceptance interval for all but one of the analyzed batches and time points ( Table 1 ). A specific activity of 63 IU/µg was reached at 6 hours for batch 2 in setup C at 6 hours; for this time point, the 90% CI was outside the acceptance interval.

Content of rFVIIa remained stable across the samples used in each test system, decreasing from the reference values by <0.1 mg/vial for all samples ( Table 1 ). Reference values were between 5.2 and 5.5 mg/vial for batches 1 and 2, and 8.3 and 8.2 mg/vial for batch 3.

Product degradation was very limited compared with the reference samples, with similar levels of sample degradation seen for 12- and 24-hour samples ( Table 1 ). With a dosing interval of 6 hours, limited degradation of rFVIIa was observed, compared with the reference sample. The same levels of rFVIIa degradation were observed for samples with a dosing interval of 2 hours.

No changes in transparency, color, or presence of sedimentation in the syringe and infusion tube were observed during the 24-hour duration of the study. Some inherent particles were seen in the reconstituted rFVIIa; when analyzed by stereomicroscopy and Fourier transform infrared spectroscopy, these particles were found to consist of a combination of protein and silicon, and were observed as soft, bright, regular, and transparent.

Organic leachables were observed in concentrations below the analytical evaluation threshold level. Anionic, metal, and trace element leachables were observed in concentrations below their respective permitted daily exposures.

No microbiological growth was detected during or at the end of incubation of the batches tested.

## Discussion

Analysis of the physical and chemical stability of rFVIIa showed that the 90% CIs for the quality parameters complied with the acceptance criteria for all the analyzed batches and time points. Product degradation was very limited.

For the parameter of specific activity, batch 2 in setup C at 6 hours showed upper 90% CI outside the acceptance interval. As the parameter of specific activity is unlikely to increase during storage, the result after 6 hours is likely to be related to the analytical uncertainty of this method. The results in setup C after 24 hours complied with the acceptance criteria and were in the same level as all other setups, thereby confirming the stability over 24 hours at 25°C.

No changes in transparency, color, or presence of sedimentation in the syringe or infusion tube were observed during the 24-hour duration of the study. Some barely visible particles were seen during sampling of rFVIIa from the syringe and infusion tube at all time points. These particles were confirmed to be inherent particles by stereomicroscopy and Fourier transform infrared spectroscopy. The inherent particles did not differ in size or description from inherent particles present in rFVIIa reconstituted for single bolus infusion in a glass syringe.

With dosing intervals up to 6 hours, there was very limited degradation of rFVIIa compared with the reference sample. This shows that a dosing interval up to 6 hours is acceptable. The use of the polypropylene syringe and infusion tube with reconstituted rFVIIa for up to 24 hours did not result in the leaching from the test system into the drug product.

This study verified that the reconstituted rFVIIa drug product remains sterile for 24 hours, provided it is not contaminated during the reconstitution procedure. As the product formulation does not contain preservatives, 1 2 reconstitution and further handling must take place under aseptic conditions using appropriate aseptic techniques. Any pump capable of delivering regular, automated injections via a polypropylene syringe may be used, including hardware already in use at a hospital.

A limitation of this study is that it is in vitro only, with no clinical correlates. However, the wealth of clinical experience with rFVIIa and the use of established pump technology indicate that this is a valid option for delivery of rFVIIa, with the potential to increase convenience and reduce the time burden associated with dosing. 3

## Conclusions

The results from this in vitro stability study showed that reconstituted eptacog alfa (activated) is physically and chemically stable, with consistent specific activity, when used in an automated bolus infusion pump system for up to 24 hours at 25°C. Bolus injection intervals of 2 to 6 hours can be used without physical or chemical changes to rFVIIa. This study supports the use of an automated bolus infusion pump in the hospital setting, across all indications for eptacog alfa (activated).
